# Cannabidiol restores hematopoietic stem cell stemness in mouse through Atf2–Lrp6 axis after acute irradiation

**DOI:** 10.1002/mco2.70092

**Published:** 2025-02-13

**Authors:** Zhijie Bai, Congshu Huang, Huanhua Xu, Yuxin Wang, Zebin Liao, Pan Shen, Zhexin Ni, Chaoji Huangfu, Dezhi Sun, Yangyi Hu, Ningning Wang, Pengfei Zhang, Lei Zhou, Wei Zhou, Yue Gao

**Affiliations:** ^1^ Beijing Institute of Radiation Medicine Beijing China; ^2^ College of Traditional Chinese Medicine Henan University of Chinese Medicine Zhengzhou China; ^3^ State Key Laboratory for the Modernization of Classical and Famous Prescriptions of Chinese Medicine Jiangxi University of Traditional Chinese Medicine Nanchang China; ^4^ State Key Laboratory of Kidney Diseases Chinese PLA General Hospital Beijing China

**Keywords:** Atf2, cannabidiol, hematopoietic stem cell, radiation injury

## Abstract

Bone marrow serves as the residence of hematopoietic stem cells and is recognized as one of the most radiosensitive tissues. Exposure to acute radiation leads to severe damage to bone marrow hematopoiesis which can be fatal, while few clinically applicable medication or specific therapeutic targets have been discovered. In this study, we found that the administration of cannabidiol significantly enhanced individual survival and restored the reconstitution capacity of bone marrow hematopoietic stem cells within 14 days after irradiation. Single‐cell RNA sequencing analysis demonstrated that the expression levels of genes associated with stemness along with Wnt and BMP signaling pathways were restored by the cannabidiol treatment through the upregulation of *Atf2*, a transcription factor possessing multifunctional properties. *Atf2* upregulation induced by cannabidiol treatment potentially upregulated the expression of *Lrp6* to improve the stemness of hematopoietic stem cells. Further functional experiments validated the crucial role of *Atf2* in regulating multilineage differentiation potential of bone marrow hematopoietic stem and progenitor cells. Overall, our findings provide evidence for a promising radioprotective function of cannabidiol and *Atf2* as a candidate therapeutic target for acute radiation‐induced hematopoietic injury, thereby paving the way for future research in the field.

## 1 INTRODUCTION

Hematopoietic cells play crucial roles in vertebrates, including oxygen transport, organogenesis, immune defense, and maintenance of homeostasis.^1^, ^2^, ^3^ As a kind of pluripotent stem cell that can differentiate into all blood lineages, hematopoietic stem cells (HSCs) are regarded as the source of both hematopoietic and immune system in adult vertebrates and seed in bone marrow.^4^ Inhabiting in an environment with relatively low oxygen level, over 90% of HSCs remain quiescent to preserve their stemness while others enter cell cycle to self‐renew or differentiate to downstream hematopoietic and immune progenitors.^5^, ^6^ Under circumstances like hemorrhage, HSCs promptly initiate differentiation programs for blood cell replenishment, which is essential for maintaining organ function and individual survival.^7^

When exposed to acute irradiation, molecules in bone marrow components including HSCs are ionized, generating elevated radicals and reactive oxygen species (ROS). The increased ROS level further causes DNA damage and induces apoptosis, cell‐cycle arrest, senescence, necrosis, and cancer.^8^, ^9^, ^10^ Though proliferating populations are more susceptible to irradiation‐induced injury, a persistent myelosuppression or bone marrow failure occurs as a result of HSC injury after exposure to a high‐dose total body irradiation since HSCs are highly sensitive to irradiation.^11^, ^12^ Acute irradiation not only triggers cell death or apoptosis in HSCs but also facilitates the differentiation of HSCs, ultimately leading to functional HSC exhaustion.^13^, ^14^ Additionally, acute irradiation exerts a complex influence on hematopoietic niche cells like osteoblasts and endothelial cells to disrupt extrinsic physiological signals that maintain the HSC function. This disruption further delays hematopoiesis recovery.^15^ Therefore, it is crucial to develop countermeasures for preventing or treating HSC injuries caused by irradiation.

Several agents were reported to exhibit radioprotective effects on the hematopoietic system, with amifostine being one of the most effective chemical agents. It functions as a broad‐spectrum cytoprotective agent by reducing ROS levels and preventing apoptosis of hematopoietic cells.^16^, ^17^ Within half an hour of entering the body, amifostine is rapidly dephosphorylated by alkaline phosphatase into WR‐1065, a radical‐scavenging sulfhydryl metabolite that quickly consumes oxygen upon oxidation.^18^ *N*‐acetyl‐l‐cysteine and glutathione have been shown able to protect HSCs from oxidative stress and promote the recovery of hematopoiesis when administered before radiation or immediately following radiation exposure.^19^, ^20^ Other agents such as benzydamine, glutamine, statins, vitamins, flavonoid compounds, and grapeseed extract have also been reported to possess radioprotective properties, as reviewed by Wei et al.^15^ However, the application of these agents may be limited due to uncontrollable side effects at high doses or lack of validation in animal models.

Cannabidiol (CBD) is the primary non‐psychoactive chemical from Cannabis Sativa and an important component of hemp seed, a traditional Chinese medicine with a long history of application. Based on its multiple function, CBD has been investigated in fields of nervous system diseases, analgesic therapy, aging, anti‐tumor therapy, and so on.^21^, ^22^, ^23^, ^24^, ^25^, ^26^, ^27^ In this study, we first assessed the potential role of CBD in preventing and treating acute irradiation‐induced hematopoietic injury in bone marrow. Using single‐cell RNA sequencing and functional assay, we dissected molecular alterations and potential mediator under CBD‐treatment which led to the facilitated recovery of the HSC function. Collectively, this work strongly supports the therapeutic application of CBD in irradiation‐induced bone marrow hematopoietic injury and highlights *Atf2* as a promising therapeutic target herein.

## 2 RESULTS

### 2.1 Cannabidiol facilitated the recovery of bone marrow hematopoietic stem/progenitor cells under acute irradiation

**FIGURE 1:**
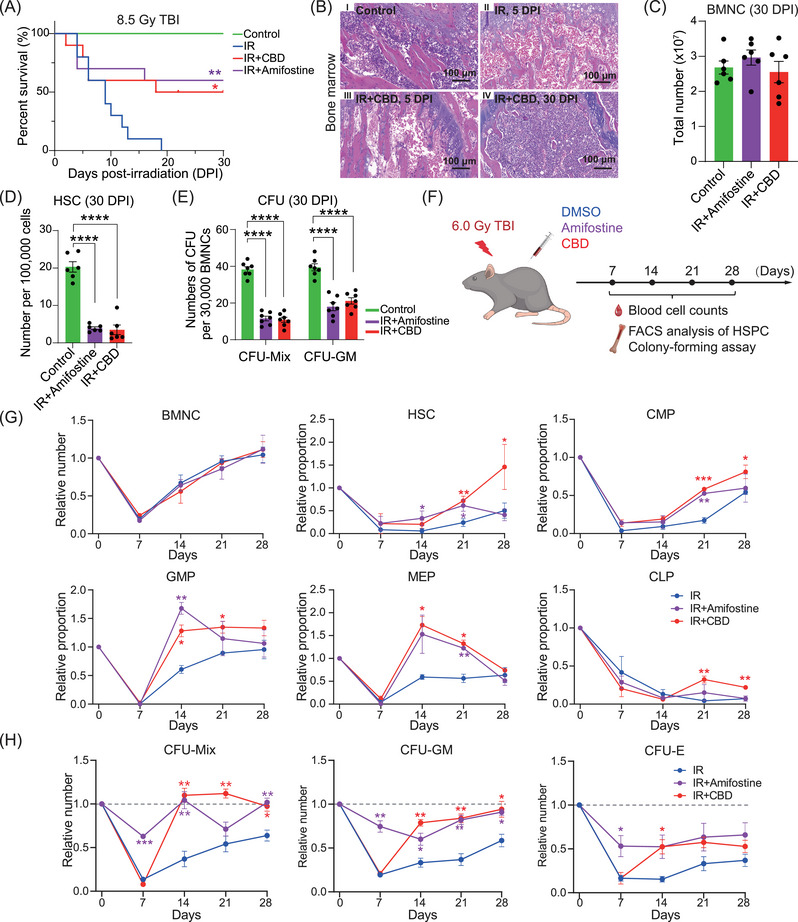
CBD facilitated the recovery of HSPCs after acute irradiation. (A) Survival plot showing 30‐day survival of mice from indicated groups. Significance test was performed between mice from IR group and other two groups. *p < 0.05, **p < 0.01 (Log‐rank test, *n* = 20). (B) Representative hematoxylin‐eosin images of bone marrow from control group (panel I), IR group at 5 days post‐irradiation (DPI, panel II), IR+CBD group at 5 DPI (panel III), and IR+CBD group at 30 DPI (panel IV). The scale bar represents for 100 µM. (C) Numbers of BMNCs in indicated groups at 30 DPI (*n* = 6). Data are presented as mean ±  standard error of the mean (SEM). (D) The ratio of phenotypical hematopoietic stem cells detected by flow cytometry in indicated groups at 30 DPI (*n* = 6). Data are presented as the mean ± SEM. ****p < 0.0001 (*t*‐test). (E) The number of colony‐forming unit (CFU) in indicated groups at 30 DPI (*n* = 7). Data are presented as mean ± SEM. ****p < 0.0001 (*t*‐test). (F) The schematic diagram of experimental design under 6.0 Gy total body irradiation. Dimethyl sulfoxide (DMSO), Amifostine and CBD were administered as described in Section 4 before or around irradiation. The blood cell counts, the number of phenotypical hematopoietic stem/progenitor cells, and CFUs in bone marrow were analyzed every 7 days within a month. (G) The dynamics of the number of BMNCs, phenotypical HSPCs in indicated groups within a month post‐irradiation. The relative number was obtained by dividing the indicated number by the corresponding number in the control group (*n* = 5). Data are presented as mean ± SEM. Statistical significance test was performed between data from other groups and data from the IR group: *p < 0.05, **p < 0.01, ***p < 0.001 (*t*‐test). (H) The dynamics of the numbers of CFUs in the bone marrow from indicated groups within a month post‐irradiation. The relative number was obtained by dividing the indicated number by the corresponding number in the control group (*n* = 5). Data are presented as mean ± SEM. The statistical significance test was performed between data from other groups and data from the IR group: *p < 0.05, **p < 0.01, ***p < 0.001 (*t*‐test). CBD, Cannabidiol; HSPC, hematopoietic stem/progenitor cells; BMNC, bone marrow nucleated cell.

We performed large‐scale screening on traditional Chinese medicine extracts or compound components that present the potential radio‐protective activity. Among the dozens of samples tested, CBD increased the 30‐day survival rate of mice from 0% to over 50% under lethal 8.5 Gy γ‐radiation (Figure 1A). We therefore focused on CBD for its potential radioprotective function. A complete bone marrow defect at 5 days post‐irradiation (DPI) was depicted by nearly absent bone marrow nucleated cells (BMNCs) but was recovered to a normal level at 30 DPI in mice treated with CBD (Figures 1B,C and S1A). Based on a set of widely recognized phenotype of hematopoietic stem/progenitor cells (HSPCs),^28^ the flow cytometry analyses illustrated similar number of HSCs (defined as Lin^−^Sca1^+^Kit^+^CD48^−^CD150^+^) in mice treated with CBD and amifostine (Figures 1D and S1B–G). The numbers of colony‐forming unit mixture (CFU‐Mix) and colony‐forming unit granulocyte/monocyte (CFU‐GM) in mice treated with CBD and amifostine were also comparable (Figure 1E).

Capturing the recovery dynamics of hematopoietic populations is essential for determining whether CBD can improve the recovery of HSPCs. Therefore, we applied radiation at 6.0 Gy which allowed mice treated with solvent control to survive within 30 days (Figure 1F). No significant differences in the numbers of peripheral blood cells were observed until 28 DPI when the number of neutrophils and monocytes in mice treated with CBD and amifostine largely exceeded those in mice treated with solvent control (Figure S1H). Among all the time points, irradiation‐induced hematopoietic injury in bone marrow was most severe at 7 DPI (Figure 1G). CBD significantly facilitated the recovery of granulocyte–monocyte progenitors (GMPs) and megakaryocyte‐erythroid progenitors (MEPs) since the second week and facilitated the recovery of HSCs, common myeloid progenitors (CMPs), and common lymphoid progenitors (CLPs) since the third week post‐irradiation (Figures 1G and S1I).

Functional assay and phenotypical analyses generally showed a distinct pattern of recovery of functional HSPCs in mice treated with CBD and those treated with amifostine. Specifically, the number of CFUs and Lin^−^Sca1^+^Kit^+^ (LSK) populations in mice treated with amifostine showed relatively attenuated decrease at 7 DPI, consistent with the previous reported pan‐cytoprotective function of amifostine^16^, ^17^ (Figures 1H and S1J). Distinctively, CFU numbers in mice treated with CBD decreased dramatically to a similar level with those in mice treated with solvent control at 7 DPI but returned rapidly to a normal level at 14 DPI (Figure 1H). This was consistent with the enhanced recovery of LSK populations in mice treated with CBD (Figure S1K, L). These data collectively demonstrated that CBD significantly facilitated the recovery of functional HSPCs within 2 weeks post‐acute irradiation.

### 2.2 Cannabidiol attenuated oxidative stress and restored hematopoietic stem cells stemness within 14 DPI

**FIGURE 2:**
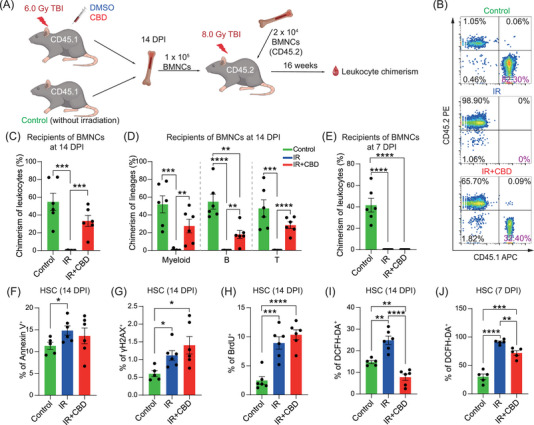
CBD alleviated oxidative stress and restored the function of HSC within 14 DPI. (A) The schematic diagram depicting the competitive transplantation assay of bone marrow nucleated cell (BMNC). At 14 DPI, 1 × 10^5^ BMNCs from CD45.1/CD45.1 mice of three groups were transplanted into lethally irradiated CD45.2/CD45.2 recipients with 1 × 10^5^ competitor BMNCs by intravenous injection. At 16 weeks post‐transplantation, chimerism in CD45^+^ leukocytes were detected by flow cytometry. (B) Representative flow cytometry plots showing leukocyte chimerism in recipients of indicated groups. The percentage of donor‐derived populations in leukocytes was showed in purple. (C) Bar plot showing the chimerism of leukocytes at 16 weeks post‐transplantation in recipients transplanted by mice from three groups (*n* = 6). Data are presented as mean ± SEM. ***p < 0.001 (*t*‐test). (D) Bar plots showing the chimerism of myeloid, B and T cell lineages at 16 weeks post‐transplantation in recipients transplanted by mice from three groups (*n* = 6). Data are presented as mean ± SEM. **p < 0.01; ***p < 0.001; ****p < 0.0001 (*t*‐test). (E) Bar plot showing chimerism in recipients of HSCs at 7 DPI from indicated groups (*n* = 6). Data are presented as mean ± SEM. ****p < 0.0001 (*t*‐test). (F) Bar plot showing the percentage of Annexin V positive populations in HSCs of indicated groups at 14 DPI (*n* = 5 for control, *n* = 6 for IR and IR+CBD). Data are presented as mean ± SEM. *p < 0.05 (*t*‐test). (G) Bar plot showing the percentage of γH2AX‐stained populations in HSCs of indicated groups at 14 DPI (*n* = 5 for control, *n* = 6 for IR and IR+CBD). Data are presented as mean ± SEM. *p < 0.05 (*t*‐test). (H) Bar plot showing the percentage of BrdU positive cells in HSCs in indicated groups at 14 DPI (*n* = 6). Data are presented as mean ± SEM. ***p < 0.001; ****p < 0.0001 (*t*‐test). (I) Bar plot showing the percentage of DCFH‐DA positive cells in HSCs in indicated groups at 14 DPI (*n* = 5 for control, *n* = 6 for IR and IR+CBD). Data are presented as mean ± SEM. **p < 0.01; ****p < 0.0001 (*t*‐test). (J) Bar plot showing the percentage of DCFH‐DA positive populations in HSCs from indicated groups at 7 DPI (*n* = 5). Data are presented as mean ± SEM. **p < 0.01; ***p < 0.001; ****p < 0.0001 (*t*‐test). CBD, Cannabidiol; HSC, hematopoietic stem cells; DPI, days post‐irradiation.

The capacity of multi‐lineage reconstitution for no less than 4 months in recipients is the gold standard for HSC stemness.^29^ Given the substantially increased numbers of CFUs at 14 DPI induced by CBD, we investigated the function of HSCs at this time through competitive transplantation assay (Figure 2A). Recipients with more than 1% leukocytes derived from donor cells were recognized as repopulated. Surprisingly, all recipients of BMNCs from CBD‐treated mice showed reconstitution level similar with that in recipients of normal BMNCs at 16 weeks post‐transplantation while BMNCs from mice treated with solvent control failed to reconstitute any recipient (Figure 2B,C). Further lineage analysis confirmed successful reconstitution of all leukocyte lineages in recipients of BMNCs treated with CBD (Figures 2D and S2A). Notably, when transplanted at 7 DPI, recipients of BMNCs either treated with CBD or solvent control showed no reconstitution (Figures 2E and S2B), illustrating that the stemness of HSCs was restored by CBD treatment within the second week post‐irradiation.

Irradiation‐induced cellular injury includes increased oxidative stress, DNA damage, arrested cell cycle and enhanced apoptosis.^15^, ^30^ Our analyses revealed significantly increased proportion of BrdU^+^, DCFH‐DA^+^, Annexin V^+^ and γH2AX^+^ populations in HSCs induced by acute irradiation at 14 DPI (Figures 2F–I and S2C–G). No significant differences were observed in the proportions of BrdU^+^, Annexin V^+^, and γH2AX^+^ populations in HSCs with CBD treatment. Importantly, the DCFH‐DA^+^ proportion in HSCs and the mean fluorescence intensity (MFI) of DCFH‐DA in positive HSCs of mice treated with CBD were significantly reduced when compared to those treated with solvent control (Figures 2I and S2G), suggesting a strong anti‐oxidative function of CBD. Further, the reduced proportion of DCFH‐DA^+^ population in HSCs of IR+CBD group was also observed at 7 DPI though at a relatively lesser extent (Figures 2J and S2H). These data suggested a continuously reduced ROS level induced by CBD since early phase after irradiation, which might be the primary reason for the restored stemness of HSCs (Figure S2I).

### 2.3 Cannabidiol treatment increased the hematopoietic activity both in vitro and in vivo under non‐radiation conditions

**FIGURE 3:**
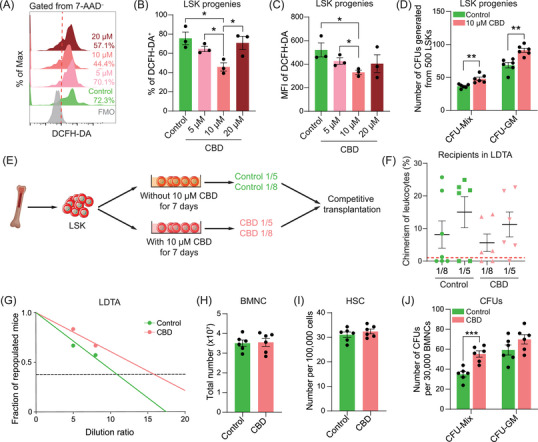
CBD treatment increased the hematopoietic activity both in vitro and in vivo. (A) Representative flow cytometry plots of DCFH‐DA staining in LSK progenies after culture with CBD at indicated concentrations for 7 days. (B,C) Bar plot showing the percentage of populations stained by DCFH‐DA in LSK progenies (B) and mean fluorescence intensity of DCFH‐DA in DCFH‐DA positive LSK progenies (C) after the 7‐day culture with CBD (*n* = 3). Data are presented as mean ± SEM. *p < 0.05 (*t*‐test). (D) Bar plot showing the number of CFUs generated from 500 LSKs in vitro in indicated groups (*n* = 6). Data are presented as mean ± SEM. **p < 0.01 (*t*‐test). (E) Schematic diagram depicting the experimental design of limiting dilution transplantation assay (LDTA). After culture for 7 days, 1/5 and 1/8 of LSK progenies were transplanted with competitor BMNCs into lethally irradiated recipients. (F) Dot plot showing the leukocyte chimerism in recipients of indicated groups at 16 weeks post‐transplantation. (G) The limiting dilution plot showing the estimated number of repopulating HSC in indicated groups. (H) Bar plot showing the number of BMNC in the bone marrow of mice with or without CBD treatment (*n* = 6). Data are presented as mean ± SEM. (I) Bar plot showing the number of phenotypical HSC in the bone marrow of mice in indicated groups (*n* = 6). Data are presented as mean ± SEM. (J) Bar plots showing the ratio of CFUs in the bone marrow of mice from indicated groups (*n* = 6). Data are presented as mean ± SEM. ***p < 0.001 (*t*‐test). CBD, Cannabidiol; HSC, hematopoietic stem cells; BMNC, bone marrow nucleated cell.

The potential impact of CBD on HSPCs under non‐irradiation conditions naturally caught our attention. At the end of a 7‐day culture with 10 µM CBD,^31^ the ratio of DCFH‐DA‐stained population and the MFI of DCFH‐DA were significantly reduced in LSK progenies (Figure 3A–C), supporting that CBD treatment was sufficient to reduce the ROS level of LSK progenies in vitro. Furthermore, colony‐forming assays further demonstrated significantly increased CFUs, both CFU‐Mix and CFU‐GM, in cultures with CBD at 10 µM (Figure 3D). As the ROS level was reported to possess pivotal influence on the function of HSCs,^15^ this result further hinted that CBD could enhance HSC activity in vitro. Limiting dilution transplantation assay at the end of culture was then performed to verify this hypothesis (Figure 3E). It can be concluded that the number of repopulating HSCs generated from 500 LSKs were increased from 11 to about 16 (Figure 3F, G), strongly suggesting that CBD can increase the number of HSCs during in vitro culture.

The in vivo influence of CBD on HSCs was subsequently evaluated. Without irradiation, CBD was administered following the same regimen applied under irradiation conditions. Fourteen days post‐administration, the concentration of white blood cells in peripheral blood of mice treated with CBD was increased, mainly contributed by increased concentration of lymphocytes (Figure S3A). The number of BMNCs showed no significant difference, neither were the ratios of phenotypical HSPCs except for an increased proportion of CMPs in CBD‐treated bone marrow (Figures 3H,I and S3B–D). Nevertheless, the frequency of CFU‐Mix was significantly increased in CBD‐treated bone marrow (Figure 3J), implying an enhanced hematopoietic activity in bone marrow treated with CBD. Since the physiological environment of HSC in bone marrow was hypoxic while in vitro culture was exposed to constant oxygen pressure, the difference in the output of in vivo and in vitro administration of CBD further indicated that CBD might increase the number of HSCs through reducing oxidative stress after acute irradiation.

### 2.4 Single‐cell RNA profiling of hematopoietic populations in bone marrow

**FIGURE 4:**
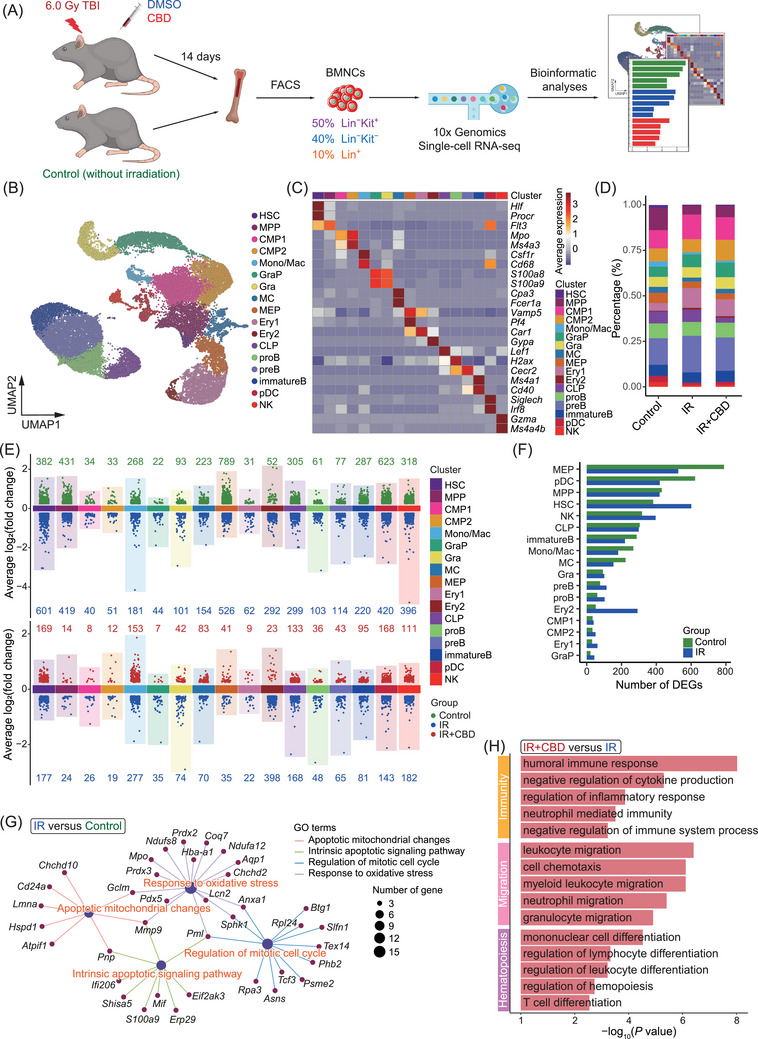
Single‐cell transcriptome profiling of BMNCs. (A) Schematic diagram of single‐cell transcriptomic profiling of BMNCs from three indicated groups. (B) Uniform Manifold Approximation and Projection (UMAP) plot showing dimensionality reduction and clustering of BMNCs. (C) Heatmap showing expression of feature markers in BMNC clusters. (D) Bar plot showing the percentage of each cluster in BMNCs from three indicated groups. The color of bars represents individual cluster and is corresponding to the color for clusters in (B). (E) Dot plots showing DEGs among groups in all clusters identified. The color of dots represents DEGs expressed in indicated groups. The number of corresponding groups was annotated on the plot in the corresponding color. (F) Bar plot showing the number of DEGs enriched in each cluster from Control and IR groups. (G) Net plot showing genes related to apoptosis, cell cycle and oxidative stress that are upregulated by acute irradiation. (H) Bar plot showing terms upregulated by the CBD treatment after acute irradiation. CBD, Cannabidiol; BMNC, bone marrow nucleated cell; DEG, differentially expressed gene.

To elucidate the molecular mechanisms underlying the enhanced hematopoietic recovery induced by CBD, we conducted single‐cell RNA profiling of BMNCs from three groups at 14 DPI (Figures 4A and S4A). After quality control, a total of 28,020 cells expressing an average of 2,544 genes were included for further analyses with no obvious batch effect (Figures 4B and S4B). With unsupervised clustering, 17 clusters were identified based on the expression of lineage‐related feature genes. These clusters covered the developmental stages from rare HSCs expressing *Hlf* and *Procr* to mature lineages including erythroid lineage (Ery 1 and Ery 2) expressing *Gata1* and *Gypa*, B cell lineage (proB, preB and immatureB) expressing *Cd19* and *Cd79a*, granulocyte lineage (GraP and Gra) expressing *Itgam* and *Ly6g*, monocyte lineage (Mono/Mac) expressing *Cd68*, natural killer cell lineage (NK) expressing *Nkg7* and plasmacytoid dendritic cell (pDC) expressing *Siglech* (Figure 4C). Lying between HSCs and mature blood cells were the progenitors including multipotent progenitor cells (MPPs) expressing *Flt3*, CLPs expressing *Lef1*, CMPs (CMP1 and CMP2) expressing *Mpo* and *Ms4a3* and MEPs expressing *Vamp5* and *Pf4* (Figures 4C and S4C,D).

The total proportion of upper stream progenitor populations corresponding to CFU‐Mix (including HSC, MPP, CMP1, and CMP2) was decreased after irradiation but recovered in mice treated with CBD to a level comparable to normal ones, consistent with the observed recovery of CFU‐Mix induced by CBD treatment (Figures 4D and 1D). Interestingly, MEP, pDC, MPP, HSC, and NK exhibited the most prominent decrease in differentially expressed genes (DEGs) following irradiation (Figure 4E,F). At the same time, the reduction of cell numbers in these five clusters was most dramatic (Figure 4D), suggesting a potential correlation between the number of downregulated genes and the decrease in quantity of specific cell type post‐irradiation. However, such correlation was not observed in the effect of the CBD treatment (Figure S4E).

Overall comparisons of BMNCs showed upregulated features related to oxidative stress, apoptosis and cell cycle induced by acute irradiation, as previously implied by the phenotypical analysis (Figures 4G and 2F–I). CBD administration caused upregulated features related to negative regulation of immunity and inflammatory response, leukocyte migration and hematopoietic cell differentiation when compared to BMNCs treated with solvent control (Figure 4H). These features implied a stimulated hematopoietic activity and alleviated damage induced by CBD on the whole‐bone‐marrow scale.

### 2.5 Cannabidiol restored stemness‐related features in hematopoietic stem cell

**FIGURE 5:**
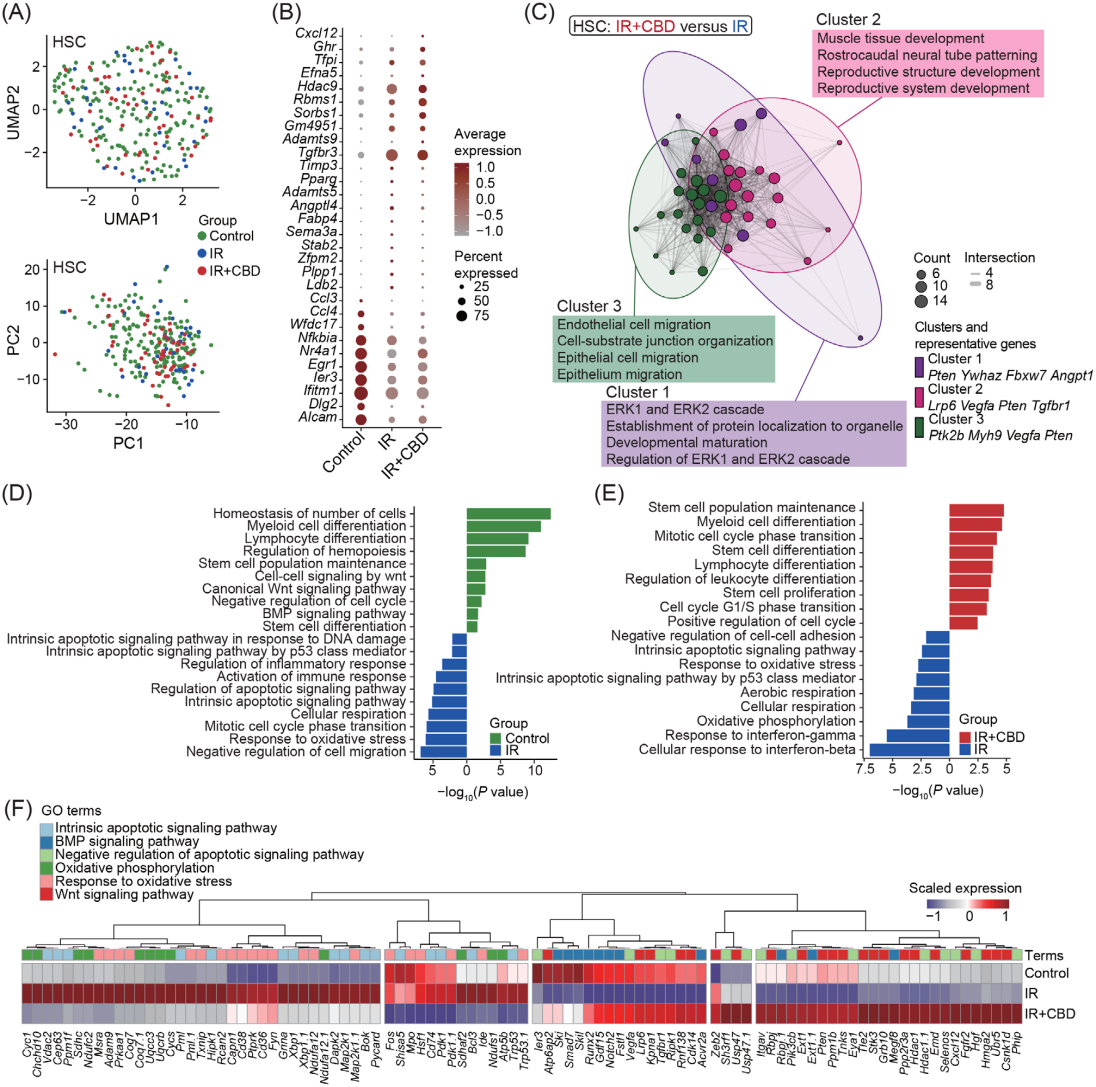
CBD treatment rescued irradiation‐induced injury and stemness in HSCs. (A) UMAP and Principal Component Analysis (PCA) plot depicting dimensionality reduction of HSCs in three indicated groups. (B) Dot plot showing expression of top 10 DEGs among HSCs from three indicated groups. (C) Enrich map showing clustered GO terms enriched by HSCs from IR+CBD group vs. those from the IR group. (D,E) Bar plots showing GO terms enriched in HSCs from control vs. IR (D) and IR vs. IR+CBD groups (E). The color of bars represents terms enriched by HSCs from indicated groups. (F) Heatmap showing the scaled expression of genes involved in specific GO terms in HSCs from indicated groups. CBD, Cannabidiol; DEG, differentially expressed gene; HSC, hematopoietic stem cells; GO, gene ontology.

In line with phenotypical analyses, the transcriptomic analysis revealed a significant reduction in HSC proportion after irradiation without an increase in the IR+CBD group (control: 0.18%; IR: 0.05%; IR+CBD: 0.06%) (Figures 1G and 4D). Additionally, the expression of homing capacity‐related genes in HSCs indicated that bone marrow hominsg capacity was not enhanced by the CBD treatment (Figure S5A). Moreover, dimensionality reduction and DEGs showed high similarity between HSCs from mice treated with solvent control and CBD (Figure 5A,B). Notably, genes associated with stemness and self‐renewal, such as *Alcam*, *Egr1*, and *Nr4a1*, ranked among the top 10 DEGs in HSCs from unirradiated mice, suggesting impacted stemness of HSCs from groups post‐irradiation^32^, ^33^, ^34^ (Figure 5B). HSCs treated with CBD were characterized by upregulated migration and development‐related features and downregulated immune and aerobic respiration‐related features compared to HSCs treated with solvent control (Figures 5C and S5B).

Physiologically, HSCs reside in a relatively hypoxic niche and the alteration of metabolic feature is related to its dysfunction.^6^ Features related to DNA damage and apoptotic signaling were upregulated in HSCs after irradiation, while those related to hematopoietic differentiation and stem cell functions downregulated (Figure 5D). The HSCs of irradiated mice were also characterized by features related to response to oxidative stress and oxidative phosphorylation when compared to unirradiated HSCs, indicating a vital role of oxidative stress in stemness loss of HSCs under acute irradiation (Figure 5D). The features enriched by HSCs treated with CBD greatly resembled those enriched by HSCs from unirradiated mice. Generally, features related to stem cells and hematopoietic differentiation were recovered in HSCs treated with CBD while those related to oxidative stress, oxidative phosphorylation and apoptotic signaling were downregulated (Figure 5E).

Gene ontology (GO) terms differentially enriched in HSCs from unirradiated mice and CBD‐treated mice after irradiation were featured by cell cycle‐related and translation‐related terms (Figure S5C,D). When compared to HSCs treated with solvent control, HSCs from unirradiated mice and CBD‐treated mice both showed upregulation of genes related to Wnt and BMP signaling (Figure 5F). These signaling pathways were reported to tightly regulate HSC development and its function. Low Wnt signaling maintains HSC proliferation and enhances HSC function, while high Wnt signaling impairs hematopoiesis and repopulation capacity.^35^ Importantly, the lack of Wnt signaling impaired the capacity of HSCs to reconstitute after transplantation.^36^ BMP signaling was also required for HSC self‐renewal since BMPR‐II deficient HSCs had impaired self‐renewal and regenerative capacity.^37^ These data illustrated on the molecular level that CBD treatment restores the stemness of HSCs within 14 DPI.

### 2.6 *Atf2* potentially mediated the stemness restoration of hematopoietic stem cells through upregulating *Lrp6*

**FIGURE 6:**
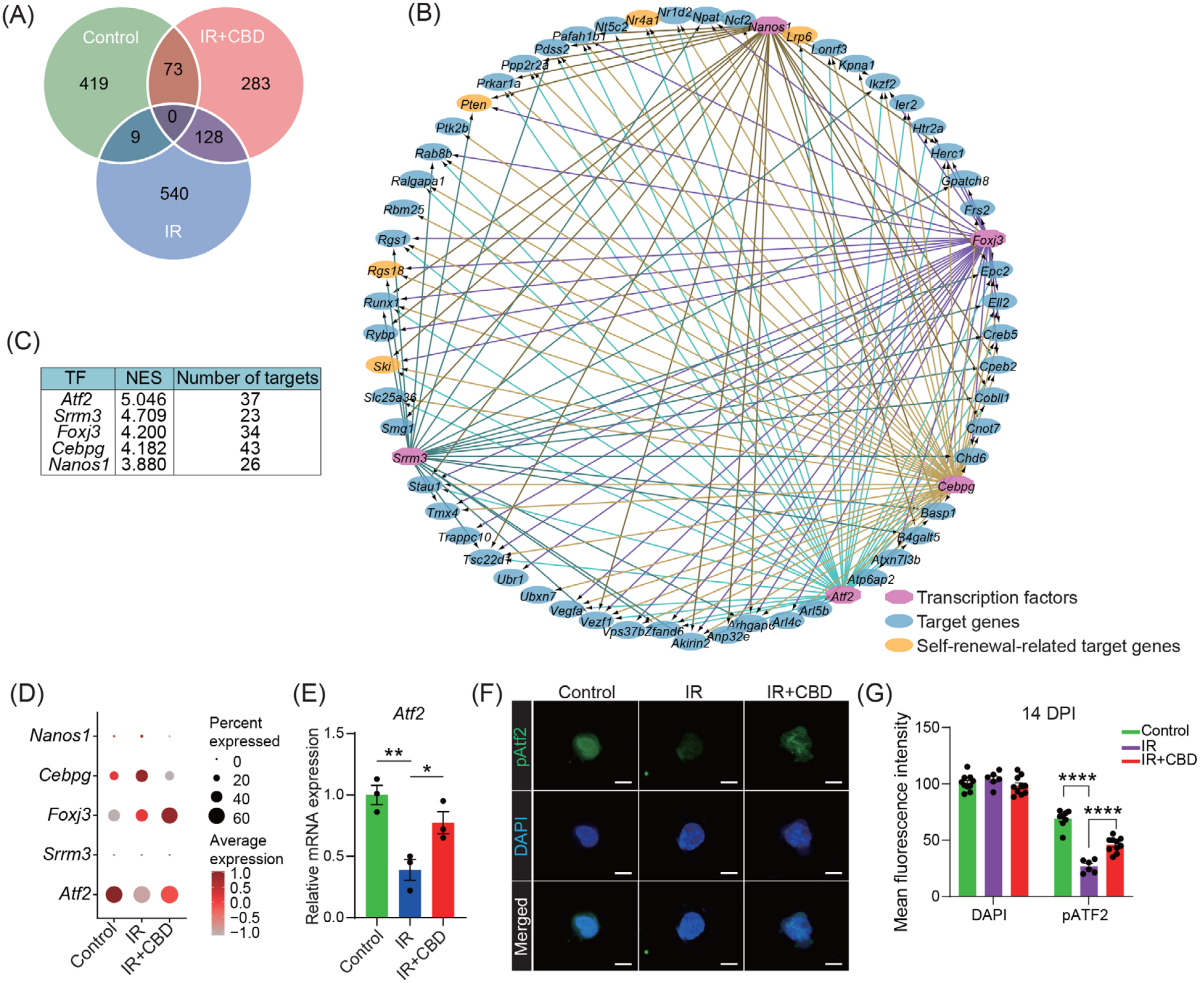
*Atf2* played crucial role in recovered stemness of HSCs induced by CBD. (A) Venn plot showing the intersection of pairwise DEGs obtained through comparing HSCs from control vs. IR and IR vs. IR+CBD groups. (B) The regulation network of predicted transcription factors and target genes. The color of edges represents regulation from indicated transcription factor. (C) Table showing normalized enrichment score (NES) and number of targets of top 5 DEGs. Transcription factors were ranked by NES. (D) The expression of top 5 transcription factors predicted by DEGs of HSCs from indicated groups. (E) The relative expression of *Atf2* in HSCs from indicated groups. *Gapdh* was used as reference gene for relative quantification of RNA expression (*n* = 3). Data are presented as mean ± SEM. *p < 0.05, **p < 0.01 (*t*‐test). (F) Representative immunofluorescence images of HSCs stained by Atf2 phosphorylated on Thr71 (pAtf2, green) and DAPI (blue) from indicated groups. The scale bars represent for 5 µM. (G) Bar plot showing the mean fluorescence intensity of DAPI and pATF2 in HSCs. Data are presented as mean ± SEM. ****p < 0.0001 (*t*‐test). CBD, Cannabidiol; DEG, differentially expressed gene; HSC, hematopoietic stem cells; DAPI, diamidino‐2‐phenylindole dye.

The pairwise DEGs revealed 73 genes shared by HSCs from unirradiated and CBD‐treated mice containing several genes related to stemness including *Nr4a1*, *Lrp6*, *Pten*, *Ski*, and *Rgs18* ^38^, ^39^, ^40^, ^41^, ^42^ (Figures 6A,B and S5E). Among the top five transcription factors (TFs) predicted to regulate these DEGs, activating transcription factor 2, encoded by *Atf2*, showed the highest enrichment score and was upregulated following the CBD treatment (Figure 6C,D). The upregulation of *Atf2* induced by CBD was further verified using quantitative RT‐PCR (Figure 6E).

It was reported that the transcriptional regulatory function of *Atf2* requires the phosphorylation on its specific amino acids including Threonine 71.^43^ We therefore detected the expression of Atf2 phosphorylated on Threonine 71 (pAtf2) in phenotypical HSCs with or without CBD administration under irradiation. Our immunofluorescence data showed that the expression of pAtf2 was also up‐regulated in CBD‐treated HSCs when compared to the IR group (Figure 6F,G). These data further supported that the role of *Atf2* in the accelerated recovery of HSC stemness induced by CBD deserved investigation.

**FIGURE 7:**
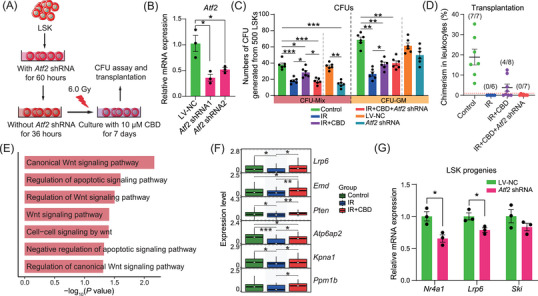
*Atf2* knock‐down counteracts CBD‐induced hematopoietic recovery. (A) Schematic diagram for in vitro functional validation of *Atf2* in CBD‐induced recovery of LSKs after irradiation. (B) Bar plot showing relative mRNA expression of *Atf2* in LSKs treated with lentivirus carrying no *Atf2* shRNA (LV‐NC) or with lentivirus carrying *Atf2* shRNA (*n* = 3). Data are presented as mean ± SEM. *p < 0.05 (*t*‐test). (C) Bar plot showing the number of CFUs generated from 500 LSKs after indicated treatment (*n* = 6). Data are presented as mean ± SEM. *p < 0.05; **p < 0.01; ***p < 0.001 (*t*‐test). (D) Dot plot showing reconstitution of recipients after 7‐day culture with CBD and *Atf2* shRNA transfection. (E) Bar plot showing enrichment of Wnt and apoptosis related features in HSCs from IR+CBD group vs. those from the IR group. (F) Box plots showing the expression of Wnt signaling‐involved genes in HSCs from three indicated groups. *p < 0.05, **p < 0.01, ***p < 0.001 (*t*‐test). (G) Bar plots showing relative expression of *Nr4a1*, *Lrp6*, and *Ski* in LSK progenies with or without *Atf2* shRNA transfection (*n* = 3). Data are presented as mean ± SEM. *p < 0.05 (*t*‐test). CBD, Cannabidiol; HSC, hematopoietic stem cells; CFU, colony‐forming unit; LSK, Lin^−^Sca1^+^Kit^+^.

Considering the relatively high expression level of *Atf2* in HSCs (Figure S5F), lentivirus containing *Atf2* shRNA was generated for in vitro knock‐down of *Atf2* (Figure 7A). The knocking down efficiency was validated by significantly downregulated expression of *Atf2* (Figure 7B) and the transfection of lentivirus carrying no shRNA exerted no influence on the number of CFUs (Figure 7C). Consistent with the in vivo data, 7‐day culture with CBD transparently recovered the number of CFU‐Mix post‐irradiation (Figure 7C). The transfection with *Atf2* shRNA specifically counteracted the increase of the number of CUF‐Mix in the IR+CBD group, implying that *Atf2* was crucial for the facilitated recovery of multipotent HSPCs induced by CBD (Figure 7C). Transplantation at the end of the culture revealed restored repopulating capacity of HSCs induced by the CBD treatment which was counteracted by knock‐down of *Atf2* (Figure 7D), illustrating the indispensable role of *Atf2* in restored repopulating capacity of HSCs induced by CBD. Surprisingly, when transfected with *Atf2* shRNA without irradiation, the number of CFU‐Mix after 7‐day culture was reduced to a relatively low level, which further illustrated the indispensable role of *Atf2* in maintaining the multipotential of physiological HSPCs (Figure 7C).

Furthermore, among GO terms upregulated upon CBD treatment, Wnt signaling was highlighted (Figure 7E). Among the DEGs contributed to Wnt signaling‐related pathways, *Lrp6* was downregulated after irradiation and restored by the CBD treatment, following an expression dynamic similar with that of *Atf2* (Figure 7F). Most importantly, in our data, *Lrp6* was predicted to be regulated by *Atf2* directly (Figure 6B). This was experimentally validated by the downregulated expression of *Lrp6* in *Atf2*‐knockdown LSK progenies (Figure 7G). Given that *Lrp6* was reported to be validly indispensable for HSC self‐renew,^40^, ^44^ these data collectively demonstrated that *Atf2* mediated the restoration of HSC stemness induced by CBD potentially through upregulating the expression of *Lrp6*.

## 3 DISCUSSION

The deficiency of the hematopoietic system induced by acute irradiation can be fatal, highlighting the crucial need for efficient molecular targets or medications. In this study, we demonstrated the stable radioprotective function of CBD under conditions of acute irradiation and observed its ability to enhance the recovery of bone marrow HSPC populations. Through competitive transplantation assays, we discovered that CBD restored the reconstitution capacity of HSCs prior to their quantification recovery, potentially attributed to a significant reduction in ROS levels. At the molecular level, CBD administration alleviated apoptotic features and oxidative stress while restoring declined Wnt and BMP signaling pathways induced by irradiation. Eventually, we identified *Atf2* as a key TF whose upregulation by the CBD treatment restored the stemness of HSCs potentially through upregulating *Lrp6*.

High‐dose irradiation causes DNA damage and exponentially increased ROS level, which further trigger apoptosis, necrosis, cell‐cycle arrest, and senescence.^8^, ^9^, ^10^ All these processes compromise the survival and function of cells. In terms of HSCs, the increased ROS level leads to more severe consequences. Our transcriptomic data showed elevated oxidative stress, enhanced oxidative phosphorylation, increased apoptotic signaling, and cell‐cycle arrest in HSCs induced by acute irradiation. Treatment with CBD significantly mitigated the ROS level of HSCs at 14 DPI, consistent with the previous reports describing CBD as anti‐oxidant agent.^21^ Nevertheless, the downregulated feature of the apoptotic process was observed in transcriptomic data but not significantly detected by the FACS analysis using Annexin V in HSCs at 14 DPI. In fact, the percentage of Annexin V positive fractions in HSCs was slightly decreased by CBD. This inconsistency between mRNA expression and protein level suggested that alterations in apoptosis may be a consequence of reduced ROS levels, thus highlighting the antioxidative function of CBD as the core mediator for the facilitated recovery of functional HSCs.

We felt it intriguing that CBD‐induced recovery of GMPs and MEPs were earlier than that of HSCs and CMPs (Figure 1G,H). Also, CBD‐induced functional recovery of HSCs was earlier than that of the number of phenotypical HSCs. Obviously, transplantation assay evaluates the repopulating function of HSC but cannot predict the biological behavior of HSCs in vivo. We hypothesized that in individuals exposed to irradiation, functionally recovered HSCs might rather differentiate into downstream populations than expand to meet the survival needs. This explains the source of increased GMPs and MEPs induced by CBD treatment, while awaits to be validated.

*Atf2*, also known as cyclic AMP response element binding protein 2 (CREB2), was a TF studied in a number of developmental and pathological conditions. Complete loss of *Atf2* leads to early post‐natal lethality characterized by the meconium aspiration syndrome.^45^ It was reported that stress‐related p38 and Jnk signal mediated the phosphorylation on Thr69/71 of Atf2, allowing its translocation into nucleus to transduce signals and activate the expression of target genes.^46^ However, in our data, the level of Atf2 phosphorylated on Thr71 was reduced in HSCs after irradiation and was increased by CBD administration. This could be explained by the data that the transcriptional expression of *Atf2* was repressed by irradiation and when CBD was administered, the expression of *Atf2* was elevated and therefore causing the level of *Atf2* phosphorylated on Thr71 increased. In this way, the upregulation of *Atf2* caused by the CBD treatment might have little, if any, correlation with the change in the ROS level.

The bone marrow niche plays crucial roles in regulating the survival, dormancy, proliferation, and differentiation of HSPCs.^47^ Irradiation‐induced injury to niche populations impairs their secretion of cytokines and signals necessary for maintaining optimal HSC function. Furthermore, the radicals and signals generated by niche populations would cause continuous damage to hematopoietic populations, especially HSCs. Unfortunately, due to rare number of niche cells in bone marrow and the methods to obtain BMNCs, no niche populations were identified in our transcriptomic data and therefore, influences of CBD on these populations and the interactions among niche populations and HSPCs were therefore not illustrated. Our further investigations will hopefully include hematopoietic niche populations.

Certainly, further investigations are warranted to elucidate additional aspects. Unlike tetrahydrocannabinol, CBD was reported to have a low affinity to classical cannabinoid receptors CB1 and CB2 and combine to GPR55, TRPV receptor and PPARγ to regulate intracellular inflammatory processes.^48^ In the context of this work, the molecular mechanisms underlying the relationship between the reduced ROS level and the upregulation of *Atf2* as well as the receptor mediating CBD‐induced hematopoietic recovery were not illustrated. Moreover, the resource of accelerated number of CMPs and MEPs at 14 DPI deserve further research. These questions will hopefully be addressed in future investigations.

## 4 MATERIALS AND METHODS

### 4.1 Mouse handling

All C57BL/6 mice were handled in specific pathogen‐free Laboratory Animal Center of Academy of Military Medical Sciences according to institutional guidelines. Male mice were applied to evaluate the radioprotective function of CBD according to the conclusions that male mice between 5 weeks to 3 months were more radiosensitive than female mice.^49^

### 4.2 Irradiation and drug administration

Mice were transferred into sterile fixing plate for Cobalt‐60 irradiation. CBD (GHEMP Biological, 13956‐29‐1) was dissolved in sterile soybean oil containing 2% DMSO (Sigma, 67‐68‐5) and was administered by intraperitoneal injection 12 h, 0.5 h prior to and 12 h, 24 h post‐irradiation at a single dose of 50 mg/kg body weight. Amifostine (MedChemExpress, 20537‐88‐6) was administered at 150 mg/kg body weight for a single injection 0.5 h before irradiation. For solvent control, mice were administered with soybean oil containing 2% DMSO.

### 4.2 Immuno‐histological staining and imaging

The immune‐histological staining of femurs, intestine and spleen were performed as previously described.^50^ For the expression of Atf2 phosphorylated on Thr71, phenotypical HSCs were sorted by flow cytometry and processed using fixing, washing and blocking regents from Beyotime biotechnology (P0098, P0106, P0102). After a whole night for blocking, cells were placed on dish (NEST, 801001) for laser confocal imaging and incubated with Atf2‐phospho‐Thr71 antibody (Abcam, ab32019, 1:500) over night. Goat anti‐rabbit IgG H&L (Abcam, ab150077, 1:1000) was subsequently incubated for 1 h and after washing. The 4′,6‐diamidino‐2‐phenylindole dye (DAPI, ThermoFisher, 62248) was subsequently stained for 10 min in the dark. Cells were imaged using full‐section imaging microscope (Zeiss, LSM980).

### 4.3 Flow cytometric analysis

Cells were washed once and resuspended in 100 µL phosphate buffered saline (PBS) containing 2% fetal bovine serum (FBS). Staining for HSPC phenotypes was performed as previously described.^28^ For apoptosis staining, Annexin V was stained together with antibodies instructing the identity of hematopoietic stem and progenitor cells. For detection of DNA damage, cells were fixed with Foxp3/Transcription Factor Staining Kit (eBioscience, 00‐5523‐00) after surface antibody staining and was subsequently stained with γ‐H2AX primary antibody (Abcam, ab22551) for 30 min. Cells were then washed once and were incubated with secondary antibody (Abcam, ab96879) at a dilution of 1/500 for 30 min before final analyses by flow cytometry. For the ROS level assessment, cells were incubated with 10 µM DCFH‐DA for 30 min. For cell cycle analysis, 200 µL BrdU at 10 mg/mL was injected peritoneally 0.5 h before sacrifice. Bone marrow was then collected and stained with BrdU antibody using BrdU Staining Kit for Flow Cytometry APC (eBioscience, 8817‐6600‐42) following instructions.

### 4.4 Colony‐forming assay

Red blood cells were depleted from bone marrow cells by a step of lysis. After being washed once and counted, 30,000 BMNCs were seeded into MythoCult M3434 (Stemcell, 03434) supplemented with 1% penicilin/streptomycin/gentamicin (Solarbio, P1410) for 7 days in 37°C incubator. Dishes were then observed under microscope for CFU identification and quantification. Colonies containing purely erythrocytes at day 3 of culture were identified as CFU‐E. At day 7 of culture, colonies containing either granulocytes, monocytes/macrophages or both were identified as CFU‐GM, while those simultaneously containing granulocytes, monocytes/macrophages and erythrocytes were identified as CFU‐Mix.

### 4.5 Competitive repopulating assay

CD45.1^+^ donor mice were treated with CBD and subjected to total body irradiation at a dose of 6.0 Gy. At 7 and 14 DPI, the donors were euthanized and BMNCs were obtained by thorough marrow flushing. Following cell counting and red blood cell lysis, 100,000 BMNCs along with 20,000 carrier BMNCs from CD45.2^+^ mice were intravenously transplanted into CD45.2^+^ male recipient mice. Immediately after transplantation, the recipients were transferred to a specific pathogen‐free barrier facility and provided with sterile water supplemented with antibiotics for a minimum duration of 1 month.

### 4.6 Single‐cell RNA‐seq library construction and sequencing

Bone marrow cells were obtained through flushing the femurs and stained with Lin and Kit as described above. Lin^−^Kit^+^, Lin^−^Kit^−^, and Lin^+^ populations from three individual mice were then sorted and mixed with a ratio of 5:4:1 for 10× Genomics single‐cell sample preparation. The single cells then processed for cDNA library construction following the standard protocol offered by 10x Genomics. Completed cDNA library was sequenced by Illumina novaseq6000 platform.

### 4.7 Single‐cell RNA‐seq data preprocessing

The fastq files generated by sequencing were processed by CellRanger (version 4.0) offered by 10× Genomics to obtain expression matrixes. Matrixes of three groups were then separately used to create Seurat object and cells meeting the following criteria were depleted from downstream analyses: (1) expressing less than 200 genes; (2) possessing more than 5% transcripts from mitochondrial genes. DoubletFinder (version 2.0.4) was then applied to predict and filter out potential doublets followed by data normalization. After quality control, Seurat object of three groups were integrated by IntegrateData offered by Seurat (version 4.4.0) using top 20 dims. Genes showing a correlation larger than 0.3 with proliferation feature genes including *Ube2c*, *Hmgb2*, *Hgn2*, *Tuba1b*, *Mki67*, *Ccnb1*, *Tubb*, *Top2a*, *Tubb4b* were removed from data. Subsequent dimensionality reduction and clustering were performed within standard framework. DEGs screening and enrichment analyses were performed using FindAllMarkers provided by Seurat and clusterProfiler (version 4.8.3), respectively.

### 4.8 Differentially expressed gene screening and enrichment analyses

DEGs were screened out by FindAllMarkers function with default parameters. Generally, 17 clusters were identified based on the expression lineage‐related feature genes. GO and Kyoto encyclopedia of genes and genomes (KEGG) enrichment analyses of DEGs were performed using clusterProfiler. For feature scoring, gene sets of specific terms were downloaded from MSigDB (https://www.gsea‐msigdb.org/gsea/msigdb). The score for gene sets was then calculated by AddModuleScore function. TF prediction and source–targets network were performed using iRegulon (version 1.3) embedded in Cytoscape software (version 3.9.1).

### 4.9 in vitro hematopoietic stem/progenitor cell culture

The basic medium for the HSPC culture was α‐MEM (ThermoFisher, 12571063) supplemented with 10% FBS, 50 ng/mL SCF (PeproTech, 250‐03), 50 ng/mL Flt3 ligand (PeproTech, 250‐31L), 50 ng/mL IL‐3 (PeproTech, 213‐13), 50 ng/mL TPO (PeproTech, 315‐14), 1% Glutamine (Sigma, 1294808) and 1% penicillin/streptomycin/gentamicin. Specific number of HSPCs were sorted and seeded into wells containing basic medium. The medium was half‐replaced every 3 days. At the end of the culture, cells were resuspended by pipetting up and down several times and collected for further colony‐forming assay.

### 4.10 *Atf2* knockdown strategy

Lentivirus carrying *Atf2* shRNA was built using services of Hanbio Tech (Shanghai, China). HSPCs were centrifuged at 400×*g* and incubated with 200 µL α‐MEM containing lentiviruses at a multiplicity of infection (MOI) of 10 for 20 min. After incubation, cell suspensions were immediately transferred to prepared culture medium for a 60‐h incubation to get maximum efficiency of virus infection. Three‐fourths to four‐fifths of the medium was then replaced with the culture medium containing no lentivirus. Cells were cultured for another 36 h without medium replacement before irradiation.

### 4.11 Statistical analyses and figure processing

Comparisons and statistical analyses among groups were performed using GraphPad Prism (version 8.3.0). *P*‐values < 0.05 were deemed to be statistically significant. Statistical analyses of single‐cell RNA‐seq data were performed using ggplot2 package (version 3.4.0). All figures were processed and assembled using Adobe Illustrator 2022.

## AUTHOR CONTRIBUTIONS

Yue Gao and Wei Zhou conceptualized this project, supervised the overall experiments and revised the manuscript. Zhijie Bai, Congshu Huang, Huanhua Xu, and Yuxin Wang performed the experiments and collected data with the help of Zebin Liao, Zhexin Ni, Ningning Wang, Pengfei Zhang, and Lei Zhou. Zhijie Bai performed bioinformatic analyses with the help of Pan Shen, Dezhi Sun, and Yangyi Hu. Zhijie Bai and Chaoji Huangfu drew the conclusions, organized figures, wrote and revised the manuscript with the help of all other authors. All authors have read and approved the final manuscript.

## CONFLICT OF INTEREST STATEMENT

The authors declare no conflicts of interest.

## ETHICS STATEMENT

Mouse manipulations were approved by the Animal Care and Use Committee of Academy of Military Medical Sciences under the ethic approval number IACUC‐DWZX‐2023‐P702. No experiment involving human participants or samples was included in this work.

## Supporting information



Supporting information

## Data Availability

The raw single‐cell RNA sequence data from our study have been deposited in the Gene Expression Omnibus (GEO) under the accession number GSE253266.
