# Supplementary material for: Cannabidiol restores hematopoietic stem cell stemness in mouse through Atf2–Lrp6 axis after acute irradiation
Source: MedComm (2020). 2025 Feb 13;6(2):e70092. doi: 10.1002/mco2.70092 (PMC11822450; doi:10.1002/mco2.70092)
Supplement: Supplementary file 1 — Supporting information [file MCO2-6-e70092-s001.docx]

***Supplementary Material***

Cannabidiol restores HSC stemness in mouse through

Atf2-Lrp6 axis after acute irradiation

Zhijie Bai, Congshu Huang, Huanhua Xu, Yuxin Wang, et al.

**Contents**

1. Supplementary figures and legends

2. Supplementary methods

**1. Supplementary figures and legends**


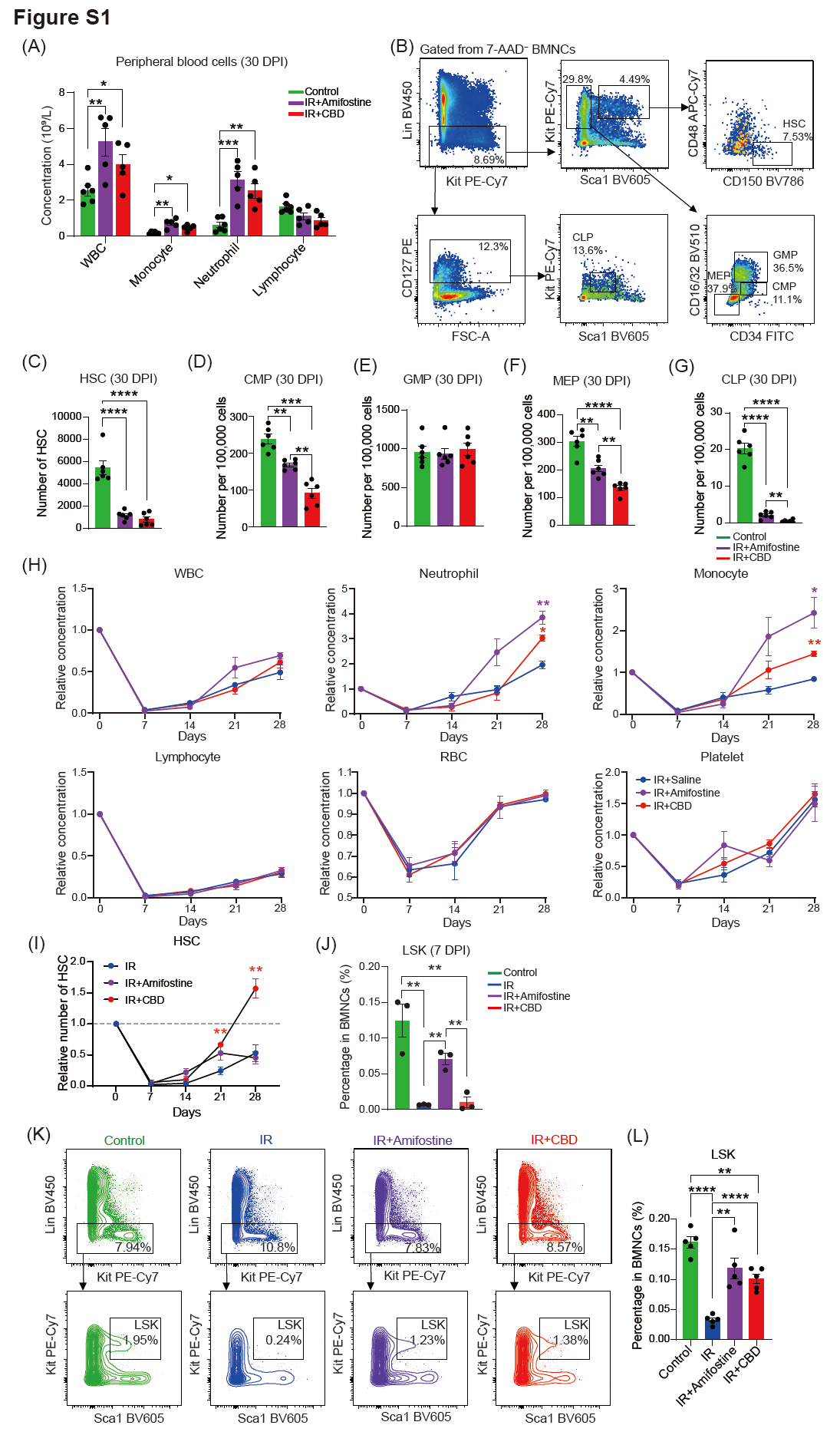


**Figure S1** **Phenotypical analyses of hematopoietic stem/progenitor cells (HSPCs) in bone marrow post irradiation.** (A) Bar plots showing concentration of blood cells in peripheral blood of mice from three groups. WBC, white blood cell (n = 6 for Control, n = 5 for IR+Amifostine and IR+CBD). Data are presented as mean ± SEM. *p < 0.05; **p < 0.01; ***p < 0.001 (t-test). (B) Representative flow cytometry plot for HSPCs including HSC, CMP, GMP, MEP and CLP at 30 DPI. (C) Absolute number of phenotypical HSCs in the bone marrow at 30 DPI after 8.5 Gy irradiation. (D-G) Ratios of CMP, GMP, MEP and CLP in indicated groups at 30 DPI (n = 6). Data are presented as mean ± SEM. *p < 0.05; **p < 0.01; ***p < 0.001; ****p < 0.0001 (t-test). (H) Relative concentrations of blood cells in peripheral blood of mice from indicated groups (n = 6). Data are presented as mean ± SEM. *p < 0.05; **p < 0.01 (t-test). (I) The dynamics of number of HSCs in bone marrow from three groups. The numbers have been normalized to that in Control group. Data are presented as mean ± SEM. **p < 0.01 (t-test). (J) Bar plot showing the percentage of LSK populations in BMNCs at 7 DPI in indicated groups (n = 3). Data are presented as mean ± SEM. **p < 0.01 (t-test). (K) Representative cytometry graphs for Lin^-^Sca1^+^Kit^+^ (LSK) populations in indicated groups at 14 DPI. (L) The percentages of LSK populations in BMNCs in indicated groups at 14 DPI (n = 5). Data are presented as mean ± SEM. **p < 0.01, ****p < 0.0001 (t-test).


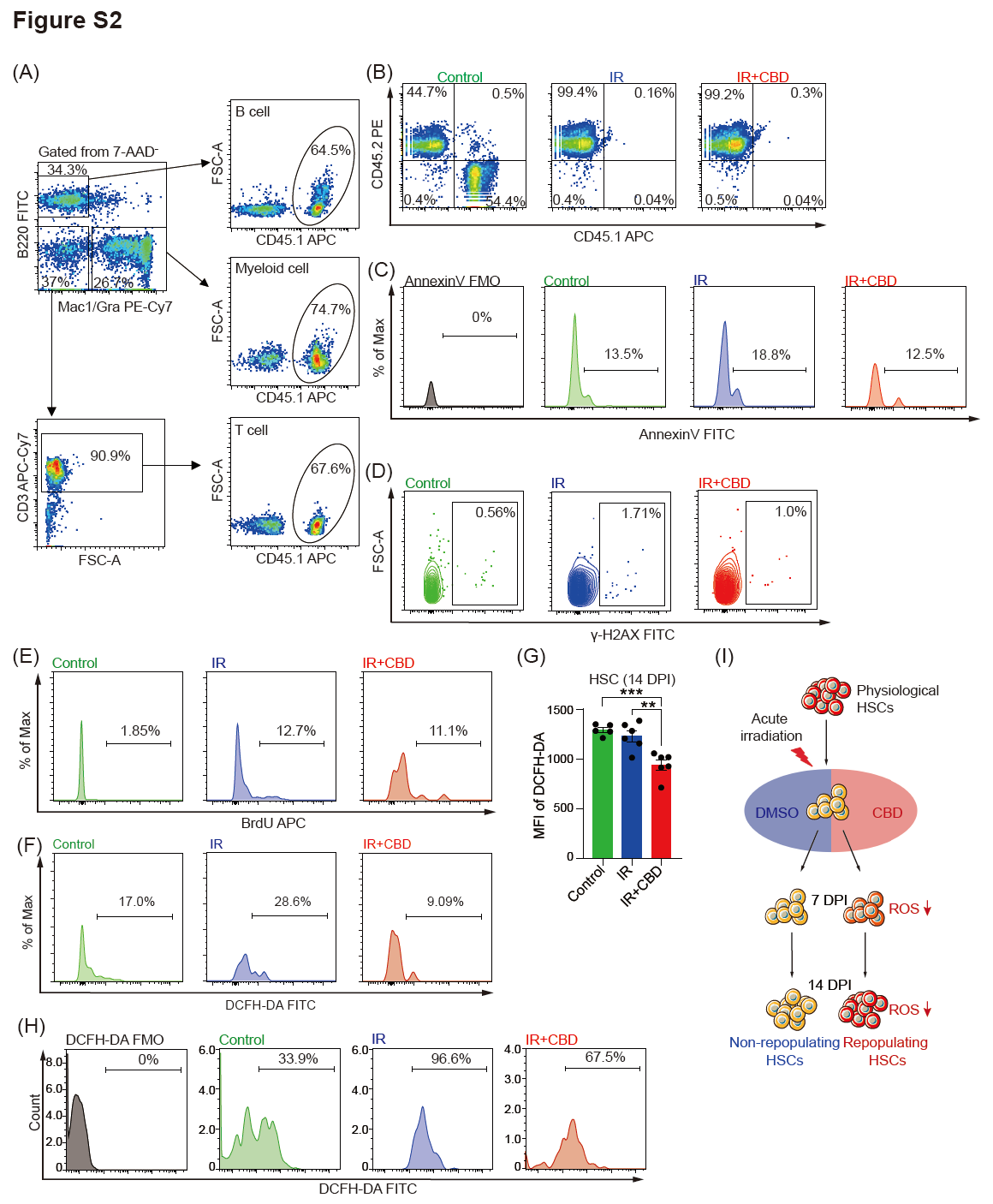


**Figure S2 CBD alleviated oxidative stress but did not recover function of HSCs at 7 DPI.** (A) Representative flow cytometry plots showing gating strategy for detecting chimerism in B cells, T cells and myeloid cells from peripheral blood of recipients at 16 weeks post-transplantation. (B) Representative flow cytometry plots for chimerism detection of leukocytes in peripheral blood of recipients receiving BMNCs at 7 DPI in indicated groups. Chimerism was detected at 16 weeks post-transplantation. (C) Representative flow cytometry plots showing staining of Annexin V in phenotypical HSCs from indicated groups at 14 DPI. (D) Representative flow cytometry plots showing staining of γ-H2AX in phenotypical HSCs from indicated groups at 14 DPI. (E) Representative flow cytometry plots showing the BrdU staining in HSCs in indicated groups at 14 DPI. (F) Representative flow cytometry plots showing the staining of DCFH-DA in HSCs in indicated groups at 14 DPI. (G) Bar plot showing the mean fluorescence intensity of DCFH-DA in DCFH-DA positive HSCs (n = 5 for Control, n = 6 for IR and IR+CBD). Data are presented as mean ± SEM. *p < 0.05; ***p < 0.001 (t-test). (H) Representative flow cytometry plots showing staining of DCFH-DA in phenotypical HSCs from indicated groups at 7 DPI. (I) Bar plot showing DCFH-DA staining of HSCs at 21 DPI.


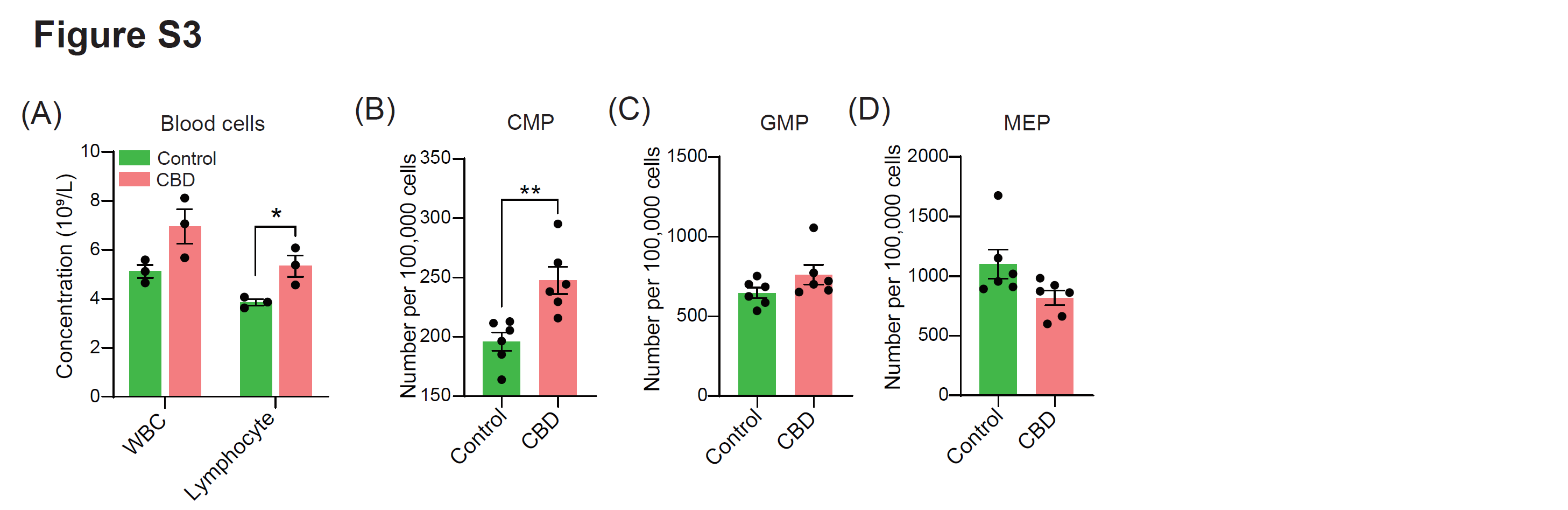


**Figure S3 Influence of CBD on hematopoietic capacity both *in vivo* and *in vitro*.** (A) Bar plots showing the change of white blood cells and lymphocytes in mice treated with or without CBD (n = 3). Data are presented as mean ± SEM. *p < 0.05 (t-test). (B-D) Bar plots showing the ratios of CMPs (B), GMPs (C) and MEPs (D) in the bone marrow at 14 days after CBD treatment (n = 6). Data are presented as mean ± SEM. **p < 0.01 (t-test).

**Figure S4** **Single-cell transcriptomic analyses of BMNCs.** (A) Flow cytometry plots showing gating strategy for BMNCs before single-cell RNA sequencing. (B) UMAP plots showing distribution of BMNCs from three indicated groups in clusters identified. (C) Dot plot showing the expression of top 5 DEGs for each cluster. (D) The expression of HSC-specific markers *Hlf* and *Procr* in all clusters from BMNCs. (E) Bar plot showing the number of DEGs enriched in each cluster from IR and IR+CBD groups.


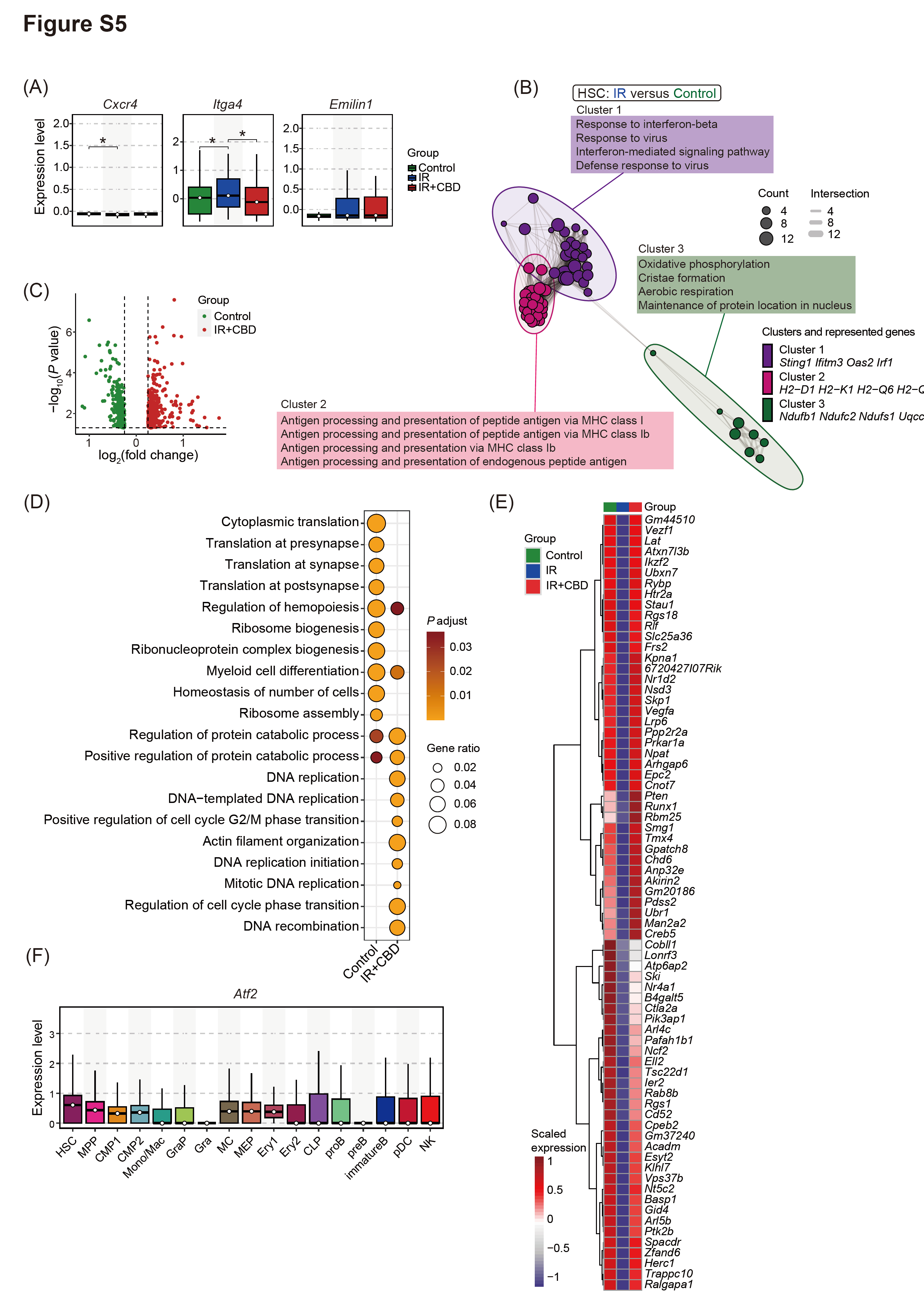


**Figure S5 Features of HSCs in three groups.** (A) The expression of bone marrow homing capacity-related genes in HSCs from three indicated groups. *p < 0.05 (t-test). (B) Enrich map showing clustered GO terms enriched by HSCs from IR group versus those from IR+CBD group. (C) Volcano plot showing DEGs in HSCs from indicated groups. (D) Dot plot showing GO terms enriched by DEGs of HSCs from indicated groups. (E) Heatmap showing genes downregulated by radiation and recovered by CBD treatment in HSCs. (F) Expression of Atf2 in clusters from bone marrow of Control group.

**2. Supplementary methods**

## **limiting dilution transplantation assay (LDTA) post-culture**

HSPCs from CD45.1/CD45.1 were obtained through FACS sorting using phenotype of LSK and cultured in vitro with or without 10 μM CBD for 7 days. For each well, 500 LSKs were seeded. At the end of culture, LSK progenies in both groups were collected and transplanted to CD45.2/CD45.2 recipientS at one-fifth and eighth equivalents, respectively. Sixteen weeks post-transplantation, blood chimerism of recipients were detected and LDTA curve was drawn.
